# Virome Sequencing of the Human Intestinal Mucosal–Luminal Interface

**DOI:** 10.3389/fcimb.2020.582187

**Published:** 2020-10-22

**Authors:** Austin Yan, James Butcher, David Mack, Alain Stintzi

**Affiliations:** ^1^Department of Biochemistry, Microbiology, and Immunology, Faculty of Medicine, Ottawa Institute of Systems Biology, University of Ottawa, Ottawa, ON, Canada; ^2^Department of Pediatrics, Faculty of Medicine, University of Ottawa, Ottawa, ON, Canada; ^3^Inflammatory Bowel Disease Centre and CHEO Research Institute, Children's Hospital of Eastern Ontario, Ottawa, ON, Canada

**Keywords:** virome, bacteriophage, phage, microbiome, gut mucosa, phageome, gut microbiome

## Abstract

While the human gut virome has been increasingly explored in recent years, nearly all studies have been limited to fecal sampling. The mucosal–luminal interface has been established as a viable sample type for profiling the microbial biogeography of the gastrointestinal tract. We have developed a protocol to extract nucleic acids from viruses at the mucosal–luminal interface of the proximal and distal colon. Colonic viromes from pediatric patients with Crohn's disease demonstrated high interpatient diversity and low but significant intrapatient variation between sites. Whole metagenomics was also performed to explore virome–bacteriome interactions and to compare the viral communities observed in virome and whole metagenomic sequencing. A site-specific study of the human gut virome is a necessary step to advance our understanding of virome–bacteriome–host interactions in human diseases.

## Introduction

The human microbiome represents a complex ecosystem of microbes, including bacteria, viruses, fungi, protozoa, and archaea. These microbes mostly reside in the gastrointestinal tract and are implicated in human health and disease, ranging from immune system development (Belkaid and Timothy, 2014) to nutrient and drug metabolism (Carmody and Turnbaugh, 2014) to involvement in conditions including obesity, inflammatory bowel disease, and cancer (Boulangé et al., 2016; Sartor and Wu, 2017; Franzosa et al., 2019). Despite major developments in microbiome research, most existing knowledge is focused on the bacteriome. The virome, consisting mostly of bacteriophages, lacks conserved marker genes and requires viral purification for cost-effective shotgun metagenomic sequencing (Garmaeva et al., 2019). Existing databases are also limited, hindering the interpretation of virome sequencing data, most of which cannot be aligned to any known viral genome (Aggarwala et al., 2017). The National Center for Biotechnology Information (NCBI) Genome Resource as of August 2020 contains over 250,000 bacterial genomes but <40,000 viral genomes, of which eukaryotic viruses are overrepresented (NCBI, 2020). Yet bacteriophages can modulate the bacteriome (Lopes et al., 2017; Hannigan et al., 2018; Clooney et al., 2019) and have a potential role in microbiome transplantation or manipulation therapies (Zuo et al., 2017; Draper et al., 2018; Lin et al., 2019; Rasmussen et al., 2020); examining the virome is essential for any comprehensive model of the host–microbiome relationship.

Recent advances in the study of the human virome include virus-like particle (VLP) purification protocols optimized for stool (Hayes et al., 2017; Shkoporov et al., 2018) and improved bioinformatic tools and databases (Roux et al., 2015; Ren et al., 2017; Gregory et al., 2020). These tools have enabled the study of the gut virome in inflammatory bowel disease (Norman et al., 2014; Clooney et al., 2019), its temporal stability in healthy adults (Shkoporov et al., 2019), and its development during infancy (McCann et al., 2018; Liang et al., 2020) and into senescence (Gregory et al., 2020). However, nearly all studies profiled stool samples, which overlook the complex biogeography of the gastrointestinal tract (Martinez-Guryn et al., 2019). Moreover, the potential for mucin–bacteriophage interactions (Barr et al., 2013) and varied microbial concentrations across the intestinal mucosa provide unique ecological pressures and niches that cannot be captured by simply sampling stool (Galley et al., 2014; Silveira and Rohwer, 2016; Duerkop, 2018; Shkoporov and Hill, 2019).

Site-specific differences in the intestinal virome have been explored in mice (Kim and Bae, 2016) and in rhesus macaques, where viromes from the terminal ileum (TI) were distinct from those of the colon and rectum (Zhao et al., 2019). Early research on the human intestinal virome characterized ileal and cecal viral particles, but these studies lacked modern high-throughput sequencing approaches (Lepage et al., 2008; Wagner et al., 2013; Hoyles et al., 2014). Two recent investigations used biopsies to study the ileal eukaryotic virome (Ungaro et al., 2018) and rectal virome (Zuo et al., 2019) in inflammatory bowel disease, the latter finding an altered virome with intestinal inflammation. Yet there has not been a focused effort to characterize the virome along the gastrointestinal tract. This information would inform our understanding of virome–bacteriome–host interactions, especially for site-specific conditions like Crohn's disease.

In this study, a protocol was developed to characterize the virome at the human intestinal mucosal–luminal interface (MLI). Aspirates obtained during endoscopy were subjected to VLP enrichment through filtering and polyethylene glycol precipitation, followed by the removal of remaining bacteria and their nucleic acids. Viral DNA was then extracted using Proteinase K and phenol–chloroform and then subjected to multiple displacement amplification (MDA) prior to sequencing. MLI aspirates can be collected at various sites along the gastrointestinal tract while providing more microbial DNA than intestinal biopsies, enabling whole metagenomic sequencing (Mottawea et al., 2019). Hence, samples were processed for both virome and metagenomic sequencing to validate the virome sequencing protocol, measure diversity within and between subjects, and explore virome–bacteriome relationships. We also compare our virome sequencing efforts with the viral signals identified in whole metagenomic sequencing. Combining both methods has been hypothesized to “improve *de novo* viral recovery” (Gregory et al., 2020).

Our samples were obtained from pediatric subjects with Crohn's disease including two individuals with active colonic inflammation, demonstrating the applicability of this protocol to investigate clinically informative samples. Thus, we provide a sample type and methodology that can be used by clinicians and researchers to study the human virome along the gastrointestinal tract.

## Materials and Methods

### Ethics Approval and Patient Recruitment

Sample collection from pediatric subjects was approved by the Research Ethics Board of the Children's Hospital of Eastern Ontario (CHEO) in Ottawa, Canada, with informed consent/assent obtained from parents and/or subjects. Samples from five patients were used in this study, which were obtained during routine endoscopy in the diagnosis and care of Crohn's disease. Subjects with infectious gastroenteritis in the past 2 months or antibiotic treatment in the past 4 weeks were excluded from this study.

### Sample Collection and Phage Spike-In

The collection of MLI aspirates has been described previously (Mottawea et al., 2019). In brief, sterile water was used to wash the bowel wall during colonoscopy to remove the loosely adherent mucous layer. The wash was then aspirated into a sterile container and stored at −80°C. Samples were obtained from three distinct sites: the terminal ileum (TI), proximal colon (PC), and distal colon (DC). Aliquots of 10 ml were used for VLP purification and viral DNA extraction; 2 ml was used for whole metagenomic DNA extraction. To estimate viral load, an exogenous phage, NCTC 12673 (Kropinski et al., 2011), was added to two samples at final concentrations of 10^5^, 10^6^, and 10^7^ plaque-forming units (pfu)/ml for VLP purification and sequencing. NCTC 12673 phage was similarly added to three samples prior to whole metagenomic sequencing at concentrations of 10^7^ pfu/ml with one of those samples also spiked at 5 × 10^7^ pfu/ml.

### Virus-Like Particle Purification and Nucleic Acid Extraction

A protocol to purify VLPs from mucosal aspirates (summarized in Supplementary Figure 1) was developed by adapting existing methods for stool (Norman et al., 2015; Shkoporov et al., 2018). Mucosal aspirates were first subjected to centrifugation twice (4,696 *g*, 10 min, 4°C) to remove debris. Samples were then sequentially filtered through 5-μm and two 0.45-μm polyvinylidene difluoride (PVDF) filters to remove host and bacterial cells. VLPs were precipitated by overnight incubation with 10% w/v PEG-8000 and 0.5 M of NaCl at 4°C and subjected to centrifugation (4,696 *g*, 20 min, 4°C) the following day. The pellet was suspended in 400 μl of saline-magnesium buffer. Remaining bacterial cells were lysed by treatment with 1 mg/ml of lysozyme (Sigma) for 30 min at 37°C followed by 0.2 volumes of chloroform (10 min, room temperature). After centrifugation (5 min, 2,500 *g*), the aqueous mixture was treated with DNase (TURBO™ DNase, Thermo Scientific) and RNaseI (Thermo Scientific) in a buffer of 1 mM of CaCl_2_ and 5 mM of MgCl_2_ for 1 h at 37°C to degrade the remaining bacterial nucleic acids. Enzymes were inactivated at 70°C for 10 min. VLPs were lysed with Proteinase K (3.2 μg/ml) in 3.2% sodium dodecyl sulfate (SDS) for 20 min at 55°C and then treated with 2.5% cetyltrimethylammonium bromide and 0.5 M of NaCl for 10 min at 65°C. Viral DNA was then extracted by adding 1 volume of phenol–chloroform–isoamyl alcohol (25:24:1, pH 6.7) to each mixture, which was vortexed and subjected to centrifugation (10 min, 8,000 *g*); this step was repeated with chloroform to remove trace phenol. Nucleic acids were purified from the aqueous layer using the DNeasy Blood and Tissue Kit (QIAGEN) and eluted in 50 μl of water. DNA was concentrated using an Eppendorf™ Vacufuge™ Concentrator to 3 μl to maximize the input DNA load for the GenomiPhi™ V2 DNA Amplification polymerase kit. MDA reactions using 1 μl of input DNA were run in triplicate and then pooled and purified with the DNeasy Blood and Tissue Kit. DNA was quantified fluorescently using the Qubit dsDNA HS Assay Kit (Thermo Fisher). This protocol was also tested on a sample of sterile water as a negative control.

### Whole Metagenome Extraction, Library Preparation, and DNA Sequencing

Whole metagenomic DNA was extracted using the FastDNA Spin Kit for DNA Isolation (MP Biomedicals), eluted in water, and quantified using the Qubit High Sensitivity dsDNA Assay Kit as previously described (Mottawea et al., 2016).

Shotgun metagenomic sequencing libraries for both virome and metagenome DNA were prepared and barcoded using the Ion Xpress Fragment Library Kit and Ion Xpress Barcode Adapters (Thermo Fisher), with sonication performed on the Covaris S220 Ultra Sonicator following the manufacturer's instructions. Libraries were visualized with the High Sensitivity DNA Kit (Agilent) on the 2100 Bioanalyzer. Samples were templated and loaded on two Ion PI Chips (virome and metagenome samples on separate chips) by an Ion Chef using the Hi-Q Chef Kit and sequenced on an Ion Proton with the Hi-Q Sequencing 200 Kit following manufacturer's instructions.

### DNA Pre-processing, Host DNA Removal, and Bacteriome Annotation

Our bioinformatic pipeline is summarized in Supplementary Figure 2. High-quality sequencing reads from the Ion Proton were trimmed using seqtk 1.2-r94 (Li, 2012) at the default error rate threshold of 0.05; reads <50 bp were also removed. Remaining reads were mapped to various databases to examine host, bacterial, and viral content using bowtie2 version 2.3.4.1 (Ziemann, 2016) with the default settings unless otherwise specified. Host and spike-in reads were detected by mapping reads to the human genome (GRCh38 with bowtie2's ultra-sensitive mode) (Schneider et al., 2017) and the NCTC 12673 genome (Kropinski et al., 2011); these reads were counted and removed from further analysis using samtools 1.7 (Li et al., 2009). Samples sequenced multiple times to assess phage-spike-in loads were also analyzed to evaluate reproducibility; these reads were then merged for subsequent analyses. Bacterial contamination and viral content were assessed by aligning reads to the cpn60 database (ultra-sensitive mode) (Vancuren and Hill, 2019), the Gut Virome Database (Gregory et al., 2020), known crAssphages (Guerin et al., 2018), and all viral genomes available on the NCBI Viral Genomes Resource (Brister et al., 2015) as of May 11, 2020 (12,194 genomes). Host-removed, whole metagenomic sequencing reads were aligned to a database of all non-redundant sequences using DIAMOND 0.9.2 (Buchfink et al., 2014) and annotated using MEGAN 6.18.3 (Huson et al., 2016). When performing bacteriome analysis, non-bacterial taxa were excluded from the whole metagenome results (an average of 0.32% of reads/sample mapped to viruses; 1.2% to eukaryotes).

### Identification of Viral Contigs in Virome and Whole Metagenomic Sequencing Data

Host and spike-in decontaminated reads from each virome sample were assembled using MEGAHIT; contigs longer than 1,000 bp from all samples were clustered using ClusterGenomes (Roux and Bolduc, 2017) at 90% identity and a minimum length of 90% of the shorter contig. Open reading frames were predicted using Prodigal v2.6.3 (Hyatt et al., 2010) in metagenomic mode. Clustered contigs were then subjected to the following viral selection criteria: positive (category 1 or 2) or circular identification by Virsorter v1.0.5 (Ren et al., 2017) in virome decontamination mode (db = Viromedb); alignment to the NCBI Viral Genomes Resource or the crAssphage database (e-value < 10^−10^) using nucleotide-nucleotide BLAST 2.9.0+ (Camacho et al., 2009); or identification of ≥3 open reading frames aligning to a 2016 database of prokaryotic viral orthologous groups (Grazziotin et al., 2017) with an e-value < 10^−5^ with at least two hits/kb of contig length, assessed by hmmscan in HMMER 3.1b2 (Eddy, 2011). Contigs ≥ 3 kb with no blastn alignments (e-value < 10^−10^) to the nt database (November 2019) were also retained. Contigs were removed if they had three or more open reading frames aligning to ribosomal proteins in the Clusters of Orthologous Groups of proteins database (Galperin et al., 2015) using blastp (e-value < 10^−10^) in BLAST 2.9.0+. Lastly, virome sequencing reads (host and spike-in reads removed) were remapped to the putative viral contigs (VCs); any contig that did not have a minimum horizontal coverage of 75% in at least one sample was likely misassembled and thus removed. These breadth of coverage statistics were calculated using samtools idxstats and mpileup (Li et al., 2009).

The same pipeline was used to identify VCs in the whole metagenome data, with Virsorter run in its default mode instead of virome decontamination mode. This set of contigs is referred to as the metagenome-derived VCs (mVCs). Both virome and metagenomic sequencing reads from each sample were mapped to the VCs and mVCs with bowtie2 and indexed with samtools for further analysis.

### Viral Contig Clustering, Taxonomic Annotation, and Bacterial Host Prediction

VCs were clustered using vConTACT2 (Bin Jang et al., 2019), which uses ClusterONE to detect and interpret protein-level relationships between contigs. Open reading frames were first generated using Prodigal in metagenomic mode, while vConTACT2 0.9.19 was run with pc-inflation and vc-inflation set to 1.5, pcs-mode set to MCL, and vcs-mode set to ClusterONE, as has been previously used in other virome studies (Clooney et al., 2019). These clusters were viewed using Cytoscape 3.7.2 with the default Perfuse Force Directed Layout using vConTACT2-derived edge-weights.

Viral annotations were performed using Demovir (Feargalr, 2019), which uses amino acid homology searches against viral references to assign viral order and family. We used the pre-built database of non-redundant viruses from TrEMBL available at figshare.com/articles/NR_Viral_TrEMBL/5822166. Bacterial hosts were predicted using WIsH 1.0 (Galiez et al., 2017), which identified the most likely bacterial host for each VC among a set of all reference and representative RefSeq bacteria genomes (*n* = 9,523) that were available in August 2020. The NCBI Viral Genome Resource (12,194 genomes) were used to generate null parameters for each host genome. If no bacterial genome was matched with a *p* ≤ 0.05, the VC was not assigned a putative host.

### Statistical Analysis

Our bioinformatic pipeline generated three main datasets: virome sequencing reads mapped to VCs, whole metagenomic sequencing reads mapped to mVCs, and whole metagenomic sequencing annotated using the non-redundant protein database that primarily characterized bacteria (B). We also mapped the virome reads to mVCs and metagenome reads to VCs when examining differences between the two viral populations.

For alignment comparisons and alpha-diversity analysis, read counts were used, with the latter subsetted to the sample with the lowest number of mapped reads (virome/VC: 1,877,966; virome mVC: 528,240; metagenome/VC: 5,358; metagenome/mVC: 5,867; bacteriome: 38,711). When stated, we applied a 75% horizontal coverage filter for each sample's VCs (Shkoporov et al., 2019); counts for contigs below this threshold were set to zero. For beta-diversity (Bray–Curtis) analysis, viral read counts were normalized to reads per kilobase per million mapped reads (RPKM), while bacteriome counts were normalized by relative abundance; additionally, viral hits or bacteria taxa that never exceeded a minimum 0.01% relative abundance in any sample were filtered out to remove very low abundance hits and potential false positives.

Analysis and plotting were performed in R 3.6.0 using phyloseq 1.30.0 (McMurdie and Holmes, 2013), reshape2 1.4.4 (Wickham, 2007), ggplot2 3.3.0 (Wickham, 2016), ggthemes 4.2.0 (Arnold et al., 2019), ggpubr (Kassambara, 2020), ggnewscale 0.4.1 (Campitelli, 2019), HMisc 4.4.0 (Harrell, 2020), and corrplot 0.84 (Wei and Simko, 2017).

## Results

### Sample Descriptions and Sequencing Statistics

Samples from five pediatric subjects (11.3–16.6 years old) with Crohn's disease were obtained between June and September 2018 at the Children's Hospital of Eastern Ontario in Ottawa, Canada (Table 1). Subjects A, B, and C had known Crohn's disease and underwent colonoscopy that was required for their ongoing medical care. Subjects D and E were treatment-naïve subjects undergoing colonoscopy for confirmation of their clinically suspected Crohn's disease.

**Table 1:** Subject and sample descriptions.

**Subject**	**Sex**	**Age (years)**	**Disease phase**	**Site**	**Site mucosal inflammation**	**Virome reads**	**Metagenome reads**
A	Male	16.6	Remission	TI	No	Insufficient DNA	Not performed
				PC	No	15,429,836^*^	22,190,375^*^
				DC	No	9,960,257	33,429,010^*^
B	Female	11.3	Remission	TI	No	Insufficient DNA	Not performed
				PC	No	25,566,766^*^	21,652,739^*^
				DC	No	Insufficient DNA	Not performed
C	Female	13.5	Flare	PC	Yes	2,002,855	Not performed
				DC	Yes	18,134,356	Not performed
D	Female	13.7	Diagnosis	PC	No	5,660,849	158,638
				DC	No	6,620,231	4,165,957
E	Male	14.1	Diagnosis	PC	Yes	6,008,391	4,932,195
				DC	Yes	6,334,986	14,043,455

*Read counts reflect quality-filtered reads*.

*TI, terminal ileum; PC, proximal colon; DC, distal colon*.

* *indicates sequencing efforts that were evaluated with the addition of an exogenous phage*.

Twelve samples were processed for virome extraction: MLI aspirates from the PC and DC were collected from all patients; MLI aspirates from the TI of subjects A and B were also available for analyses. All samples were processed for virome and whole genome sequencing (Supplementary Figure 1). Both TI samples and the DC sample from patient B did not yield sufficient viral nucleic acids for sequencing library construction (<50 ng). The remaining nine samples were subjected to virome sequencing. Shotgun sequencing of the whole metagenomes (i.e., not subject to VLP purification) of seven of these nine samples was also performed. A negative control of sterile water subjected to the virome protocol yielded no detectable quantities of nucleic acids.

For virome sequencing, a total of 98.0 million reads were obtained and subjected to quality filtering and trimming, resulting in 95.7 million high-quality reads (mean length = 198.2 bp). An average of 10.6 million reads (2,002,855–25,566,766) were obtained per sample (Table 1). For metagenomic sequencing, a total of 102.3 million reads were similarly processed, resulting in 100.6 million high-quality reads (mean length = 188.8 bp), or an average of 14.4 million reads/sample (158,638–33,429,010). Corresponding virome and metagenomic reads were matched for analysis.

### Virus-Like Particle Purification Removes Host and Bacterial Content

**Figure 1:**
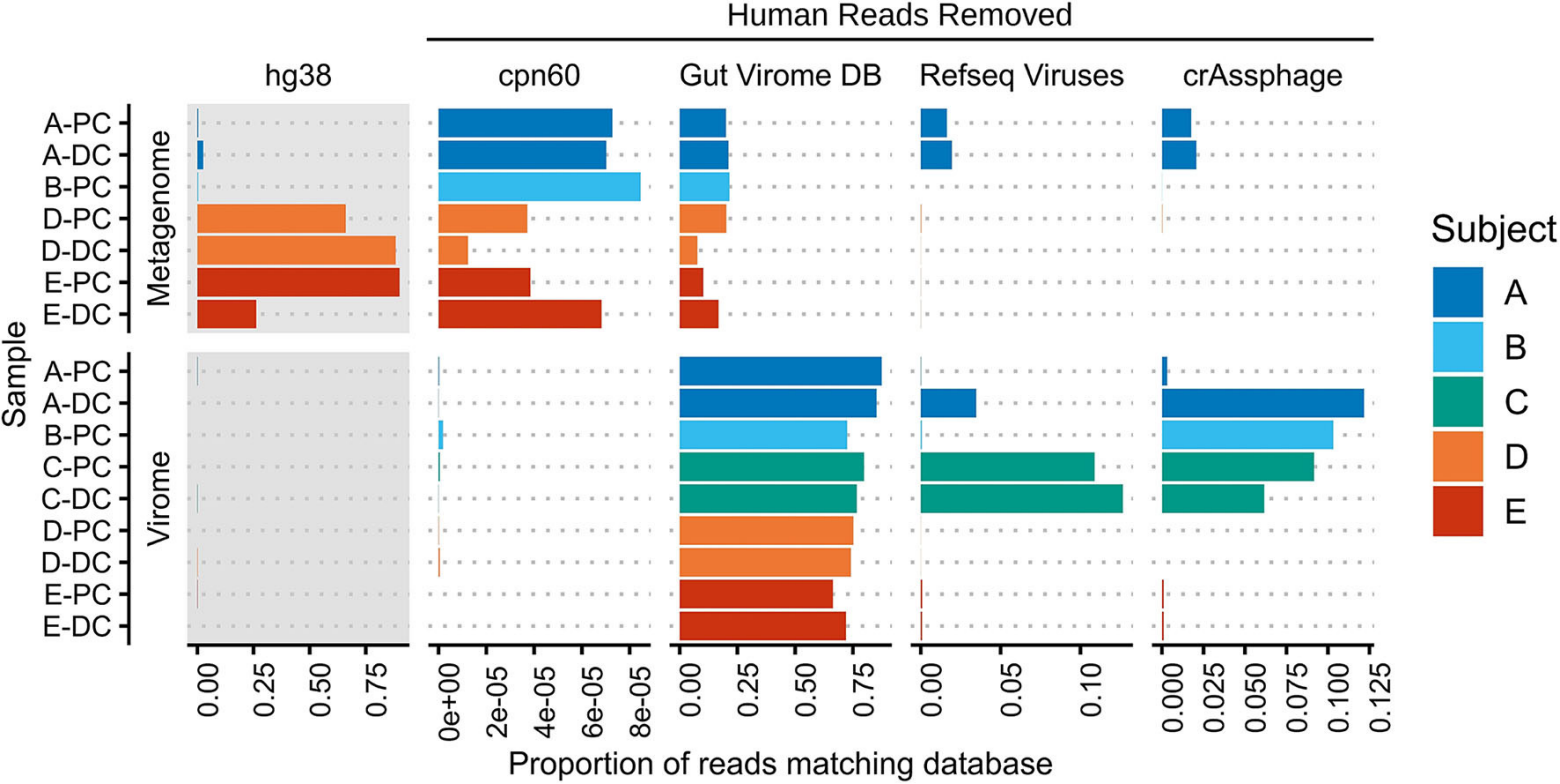
Mapping of metagenome and virome sequencing reads to human, bacterial, and viral databases. Metagenome and virome sequencing reads were mapped to the human genome (hg38), a bacterial chaperonin database (cpn60), the Gut Virome Database, all NCBI RefSeq Viruses (May 2020), and 249 crAssphage-like contigs. Bars indicate the proportion of reads aligning to each database; human read counts were only included in the gray panels, while subsequent panels were rescaled after human and phage-spike-in reads were removed. PC, proximal colon; DC, distal colon.

The alignment of virome and whole metagenomic sequencing reads to human, bacterial, and viral databases is shown in Figure 1. While host DNA is usually low in stool (<10% and often much lower) (Marotz et al., 2018; Pereira-Marques et al., 2019), an average of 39.0% of metagenome reads from the MLI samples aligned to the human genome, though varying from 0.362 to 90.0%. In contrast, an average of 0.05% (0.0028–0.17%) of virome sequencing reads aligned to the human genome, representing a mean 3,500-fold decrease in host content. This effect was most evident when host content was high in the original sample (from 5.7-fold decrease in A-PC to 20,800-fold decrease in D-PC), suggesting that host DNA is efficiently removed during the VLP purification.

The removal of bacterial DNA was assessed by aligning reads to a database of chaperonins, which are found in nearly all bacteria and have thus been used to estimate bacterial contamination (Shkoporov et al., 2018; Vancuren and Hill, 2019). The average proportion of host-removed, whole metagenome reads aligning to the cpn60 database was 0.00545%; this decreased to 0.0000401% in virome reads in the same matched samples, including two samples with no matching reads. Overall, the 136-fold decrease across the entire dataset suggests that level of bacterial contamination remaining after VLP purification is low.

Aligning virome sequencing reads to viral databases further corroborated the removal of host and bacterial DNA. An average of 76.6% of virome reads aligned to 10,673 of 33,242 viral sequences in the Gut Virome Database (66.2–87.5%) (Gregory et al., 2020), though decreasing to 41.7% (11.0–86.5%) after applying a 75% breadth of coverage filter (372 viral sequences). Thus, a significant portion of virome sequencing reads in each sample was previously identified in other virome studies, providing some evidence of a common gut viral community. These same samples had far fewer alignments to a database of all NCBI RefSeq Viruses (0.00152–12.7%), underscoring the current lack of well-annotated human gut bacteriophage genomes. Lastly, reads were mapped to a set of 249 crAss-like phage contigs, representing the most abundant human gut phage (Guerin et al., 2018); four samples had 5% or more reads matching known crAssphages. We observed a nearly 40-fold increase in crAssphage reads in A-DC compared with A-PC, suggesting site-specific differences in the human gut virome of this subject.

### Estimation of Viral Load at the Proximal Colon Mucosal–Luminal Interface

The addition of an exogenous phage, at concentrations of 10^5^, 10^6^, and 10^7^ pfu/ml, was used to estimate viral load in A-PC and B-PC samples. We used the phage NCTC 12673, a *Campylobacter jejuni* bacteriophage that was first isolated from poultry (Kropinski et al., 2011). As these patients are prescreened to ensure that they do not have infectious colitis (i.e., an active *Campylobacter* infection), this phage should be naturally absent from patient viromes. Indeed, no reads aligning to NCTC 12673 were detected in four patient viromes (A-DC, C-PC, D-DC, and E-DC); two samples (D-PC and E-DC) contained a single matching read each (<0.0001%). The remaining sample (C-DC) contained a very low abundance of reads matching NCTC 12673 (0.00015%, a 300-fold decrease from the lowest tested phage titer). Thus, NCTC 12673 is a suitable exogenous phage that could be added at 10^5^−10^6^ pfu/ml as a virome standard in patients without an active *Campylobacter* infection.

In these six phage-spiked samples, 0.044–23.3% of reads aligned to NCTC 12673, increasing linearly with phage titers (*R*^2^ > 0.99) (Supplementary Figure 3). Assuming an average phage genome size of 40 kb (Hatfull, 2008), total viral loads were estimated as 6.21 ± 0.13 × 10^8^/ml viral particles in A-PC and 1.80 ± 0.31 × 10^8^/ml viral particles in B-PC.

NCTC 12673 was also added to three MLI aspirates (A-PC, A-DC, and B-PC) and processed for whole metagenomic sequencing. No reads in the 5 × 10^7^ pfu/ml spiked sample (A-DC) and no more than one read in any 1 × 10^7^ pfu/ml spiked samples (<0.000012%) were mapped to NCTC 12673. These results suggest that exogenous, extracellular phage particles like NCTC 12673 are essentially undetectable at these spike-in concentrations using standard DNA extraction kits like the FastDNA Kit used in this study.

### Assembling and Annotating Putative Viral Contigs at the Mucosal–Luminal Interface

**Figure 2:**
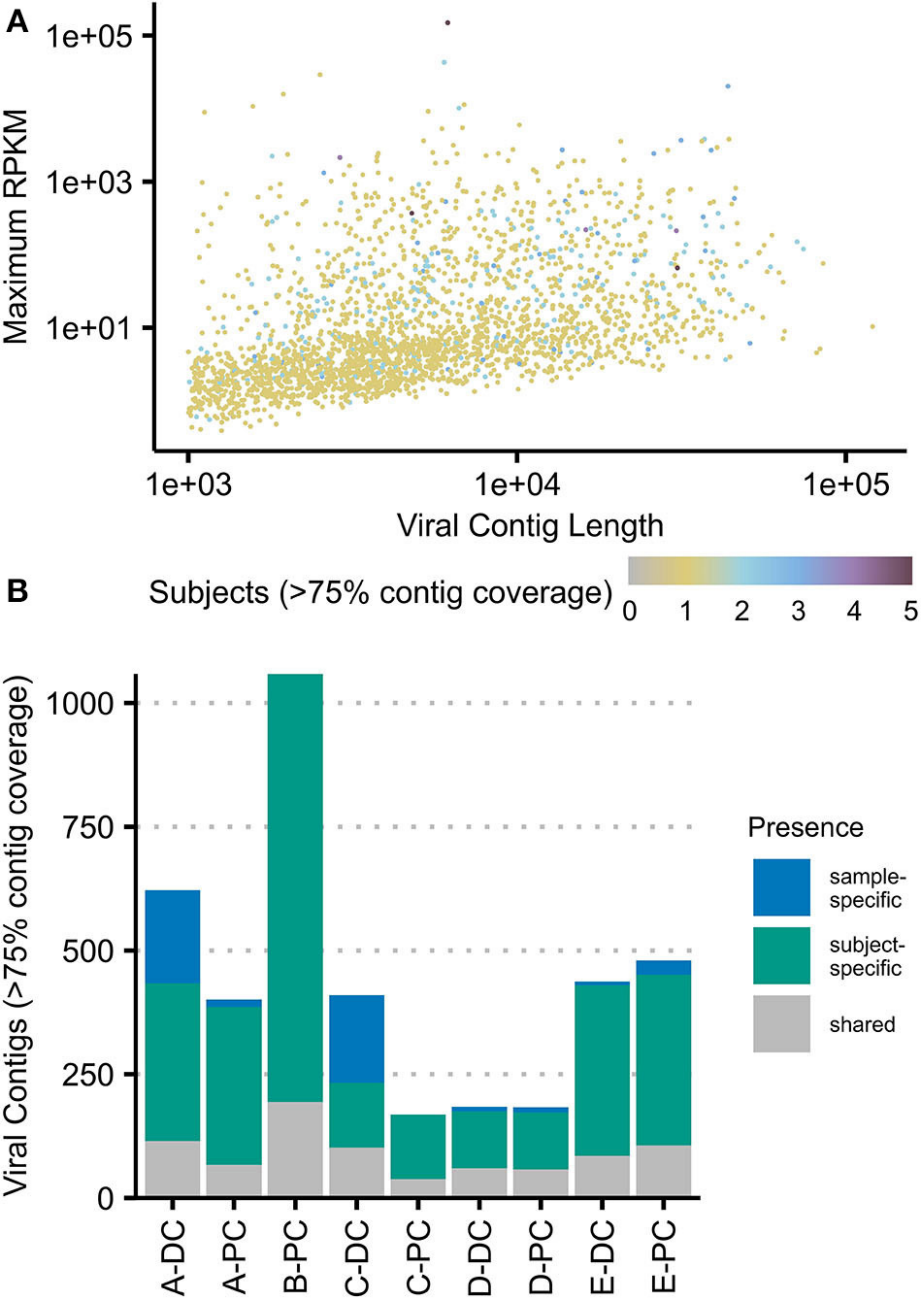
Viral contigs at the colonic mucosal–luminal interface. **(A)** All viral contigs (VCs) were plotted by their maximum observed abundance (RPKM-adjusted) vs. length. VCs were colored by the number of subjects where the contig was observed at ≥75% horizontal contig coverage. **(B)** The number of contigs present at ≥75% horizontal coverage was plotted for each sample, shaded by whether the contig was only observed in that sample, that subject, or in two or more subjects (“shared”). The same plots with each sample subsetted to two million reads are shown in Supplementary Figure 4. PC, proximal colon; DC, distal colon.

Virome sequencing reads from each sample were assembled to identify putative VCs. A total of 12,160 contigs across nine samples were pooled, clustered, and filtered for viral features, resulting in 2,511 VCs (Figure 2A). The mean contig length was 8,413 bp, ranging from 1,001 to 120,543 bp. Mapped read counts were adjusted for contig length for downstream analyses, adjusting for the 120-fold difference in VC size. Between 86.8% and 96.6% of reads from each virome sequencing sample could be mapped to these contigs. There was a correlation between unmapped virome sequencing reads to the proportion of reads aligning to the cpn60 database, suggesting that these unmapped reads could represent low-abundance, bacterial reads (Pearson correlation = 0.795, *p* = 0.0105).

At a minimum of 75% contig coverage, each sample contained an average of 648 VCs (169–1,066) with an average of 892 VCs (208–1,066) per subject. Only three VCs were present in all subjects, and only one VC was present in all samples; 44 VCs were present in at least three of five subjects. Figure 2B shows that at this level of coverage, most VCs (*n* = 2,229) were only seen in one subject; 435 VCs were also site-specific (excluding the 871 VCs only observed in B-PC). These site-specific VCs represented up to 44.6% of a sample's total observed VCs (C-DC). Breadth of coverage filtering can be confounded by sequencing depth; thus, we analyzed a rarefied dataset and found similar results (Supplementary Figure 4).

Whole metagenomic sequencing was also used for viral sequence mining following a similar bioinformatic pipeline used to assemble and filter VCs. Overall, the ratio of VCs to sequencing reads was 3.56e−5 using a metagenome-mining approach compared with 2.69e−5 in the VLP-enriched approach, representing a 32.2% increase (Table 2). A total of 3,122 mVCs with an average length of 6,233 bp were identified and compared with the virome-derived VCs. Viral families were predicted using Demovir, which annotated 62.4% of VCs and 27.9% of mVCs, as shown in Table 2. *Caudovirales*, the predominant viral order observed in the gut, represented the vast majority of annotated contigs (97.6% of VCs and 96.9% of mVCs), with *Siphoviridae, Myoviridae*, and *Podoviridae* observed in decreasing frequency. *Anelloviridae* and *Microviridae* were observed in the virome dataset (14 and 24 VCs, respectively), while only two *Microviridae* were seen among mVCs. *Herpesviridae, Iridoviridae, Mimiviridae, Poxviridae*, and *Phycodnaviridae* were only seen among mVCs, except for one *Phycodnaviridae* among VCs (compared with 17 mVCs).

**Table 2:** Virome and whole metagenome assembled viral contigs.

	**Viral contigs (VCs)**	**Metagenome-derived viral contigs (mVCs)**
Source	Virome sequencing (93.2 million reads)	Whole metagenomic sequencing (87.6 million reads)
Samples	9/9 mucosal luminal interface samples	7/9 mucosal luminal interface samples
Total viral contigs	2,511	3,122
Contigs/source reads	2.70e−5	3.56e−5
Mean contig length	8,413 bp	6,233 bp
Viral family		
*Caudovirales*		
*Myoviridae*	275	204
*Podoviridae*	90	41
*Siphoviridae*	904	430
Unassigned	285	169
*Caudovirales*		
*Other viruses*		
*Anelloviridae*	14	0
*Herpesviridae*	0	2
*Iridoviridae*	0	2
*Microviridae*	24	2
*Mimiviridae*	0	2
*Phycodnaviridae*	1	17
Unclassified	918	2,251

*Virome and whole metagenomic sequencing reads were assembled and filtered for viral properties, resulting in 2,511 viral contigs (VCs) and 3,122 metagenome-derived viral contigs (mVCs). The source read counts represent host and spike-in removed reads that were subjected to quality filtering and trimming. Contigs were annotated using Demovir*.

### The Mucosal–Luminal Interface Virome Is Subject Specific and Distinct From the Viral Community Observed in Whole Metagenomic Sequencing

**Figure 3:**
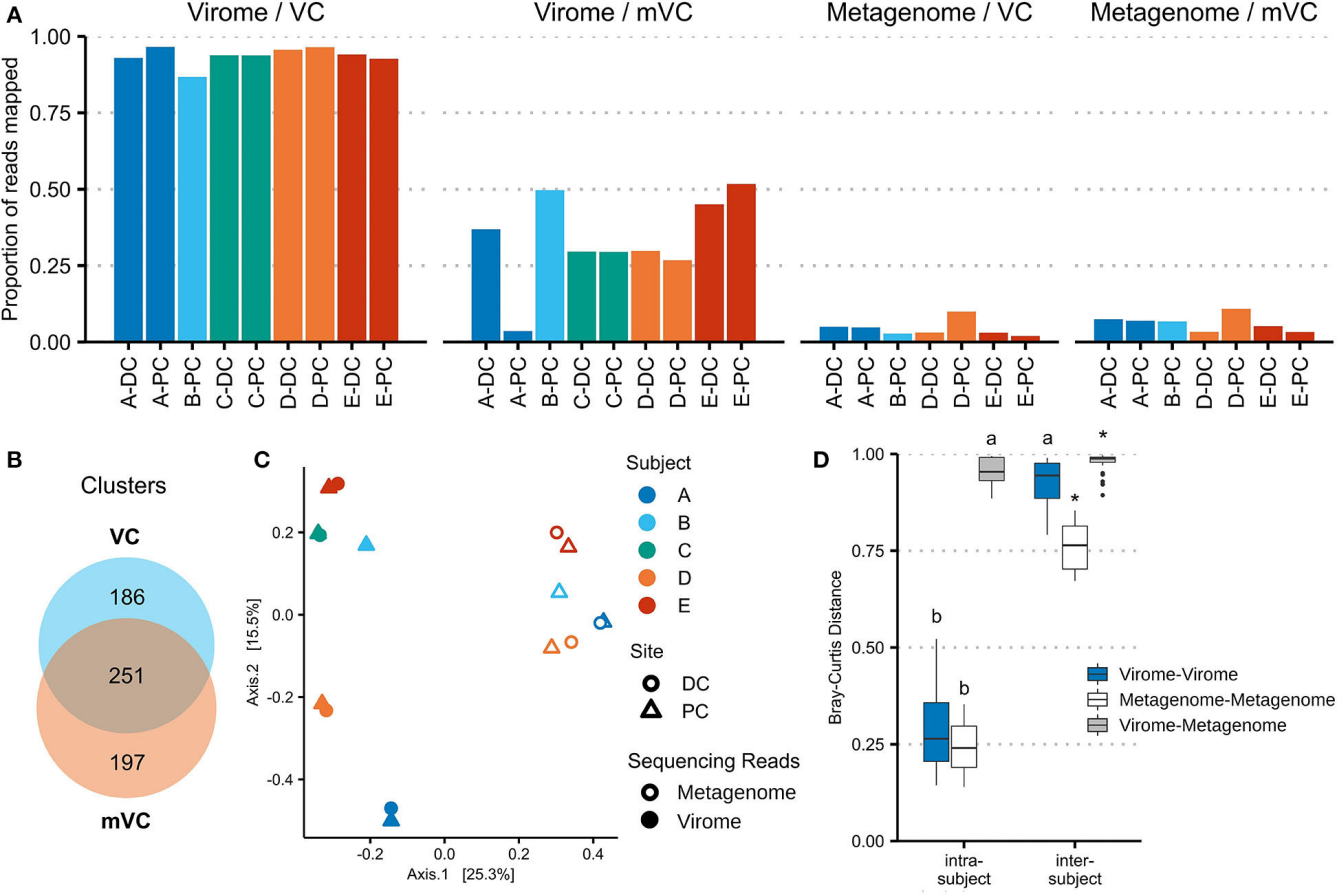
Viral contigs derived from virome and whole microbiome sequencing represent different viral populations. **(A)** Virome and whole microbiome sequencing reads were each mapped to the 2,511 virome-derived viral clusters (VCs) and 3,122 metagenome-derived viral clusters (mVCs). The proportion of reads mapped is shown for each sample. **(B)** VCs and mVCs were clustered using vConTACT2, forming 634 clusters of two or more contigs. The Venn diagram illustrates the number of clusters that include only VCs, only mVCs, or both. Network maps are shown in Supplementary Figure 6. **(C)** Virome sequencing read counts (mapped to VCs) and metagenomic sequencing read counts (mapped to mVCs) were merged using all vConTACT2-generated viral clusters. Bray–Curtis distances were calculated and plotted using principal coordinate analysis. **(D)** The Bray–Curtis distances were plotted by analysis type and compared within and between patients. *indicates a false discovery rate (FDR)-corrected *p*-value of <0.05 against every other subgroup; *a* indicates significance against all other comparisons except *a*; *b* indicates significance against all other comparisons except *b*.

Viral communities identified in virome and whole metagenomic sequencing are compared in Figure 3. Figure 3A shows the mapping of virome and metagenome reads against VCs and mVCs. On average, 93.7% of virome sequencing reads aligned to VCs, while 33.6% of reads could align to mVCs. In comparison, 6.24% of whole metagenomic sequencing reads aligned to mVCs, while 4.35% of reads aligned to VCs.

With the use of vConTACT2, the 5,633 VCs were organized into 4,473 clusters (including singletons and outliers); 634 clusters contained multiple contigs, ranging from 2 to 15 contigs. Of these clusters, 251 contained at least one VC and one mVC; 186 clusters contained only VCs, and 197 clusters contained only mVCs (Figure 3B). VC network maps are shown in Supplementary Figure 5. The viral clusters were then used to merge virome and metagenomic sequencing reads (each aligned against their respective VCs and then aggregated by viral cluster). Within the same subject, there was no significant difference in Bray–Curtis distance measured among the virus-enriched virome or the viral portion of the whole metagenome. While intersubject beta-diversity was higher overall, these distances were significantly lower in the viral portion of the whole metagenome than the virus-enriched virome (*p* = 6.2e−10). The bacteriome is also subject-specific (Supplementary Figure 6), though it is more conserved between subjects compared with VCs or mVCs.

### Technical Replicates Demonstrate Protocol Reproducibility and Virome Variation Between Locations

**Figure 4:**
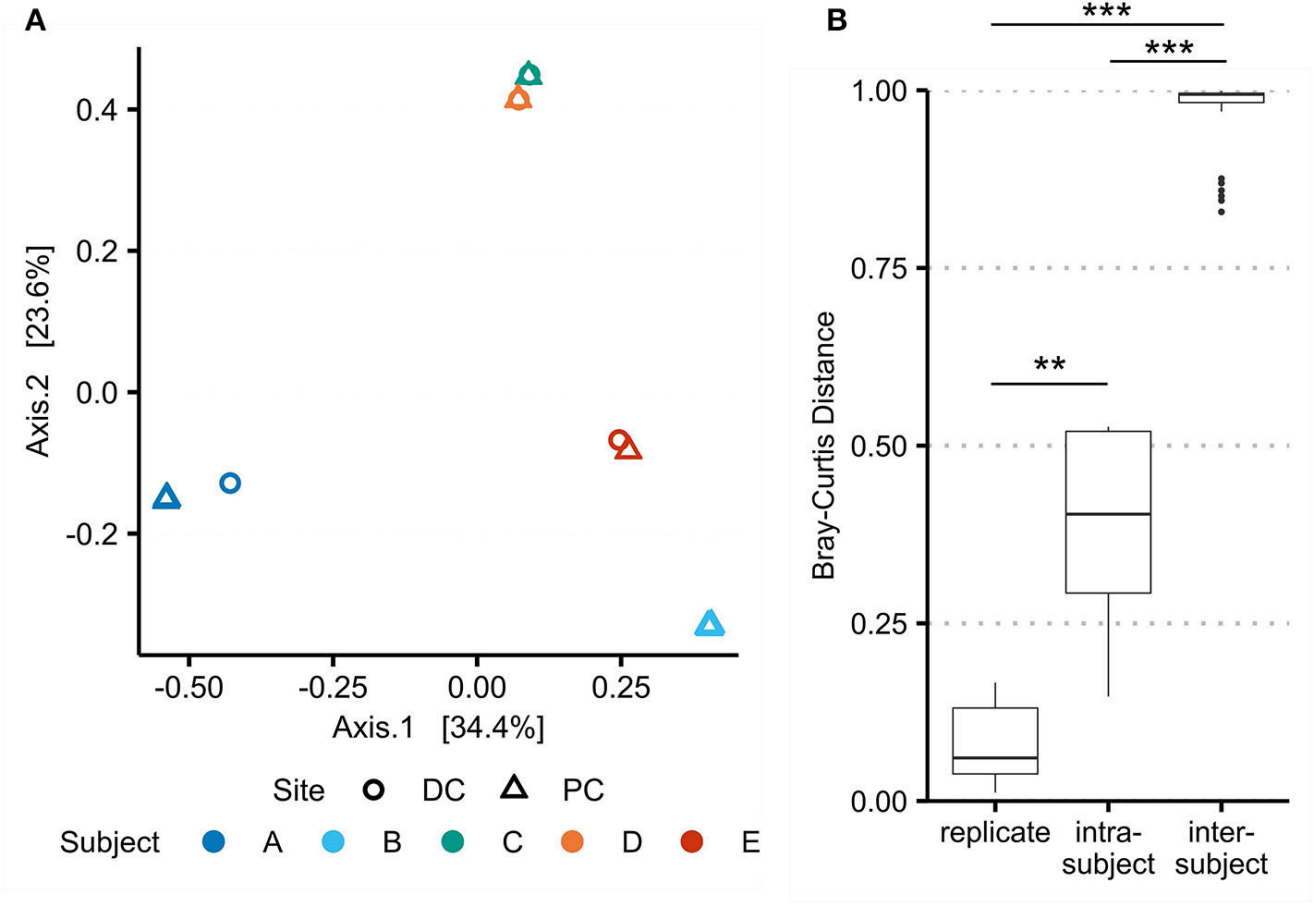
Beta-diversity between technical replicates demonstrates protocol reproducibility and intrasubject variation. Bray–Curtis dissimilarities for viral populations in the virome (as assessed using assembled viral contigs) were recalculated with A-PC and B-PC triplicates. **(A)** Principal coordinate analysis shows significant overlap of the technical replicates. **(B)** Bray–Curtis distances were compared between replicates, intrasubject samples, and intersubject samples. ** marks a false discovery rate (FDR)-corrected *p*-value of <0.01; *** <0.001.

Beta-diversity was also used to assess reproducibility of the virome protocol by comparing replicates of A-PC and B-PC (Figure 4), which were processed and sequenced in triplicate. While merged for previous analyses, aligning each replicate separately to the assembled VCs revealed a significant difference (*p* = 0.0087) in Bray–Curtis distances between PC and DC viromes (0.383 ± 0.159) compared with replicates from the same site (0.081 ± 0.063). These results demonstrate the reproducibility of the protocol while emphasizing site-specific differences in the human intestinal virome.

### Virome–Bacteriome Relationships at the Mucosal–Luminal Interface

**Figure 5:**
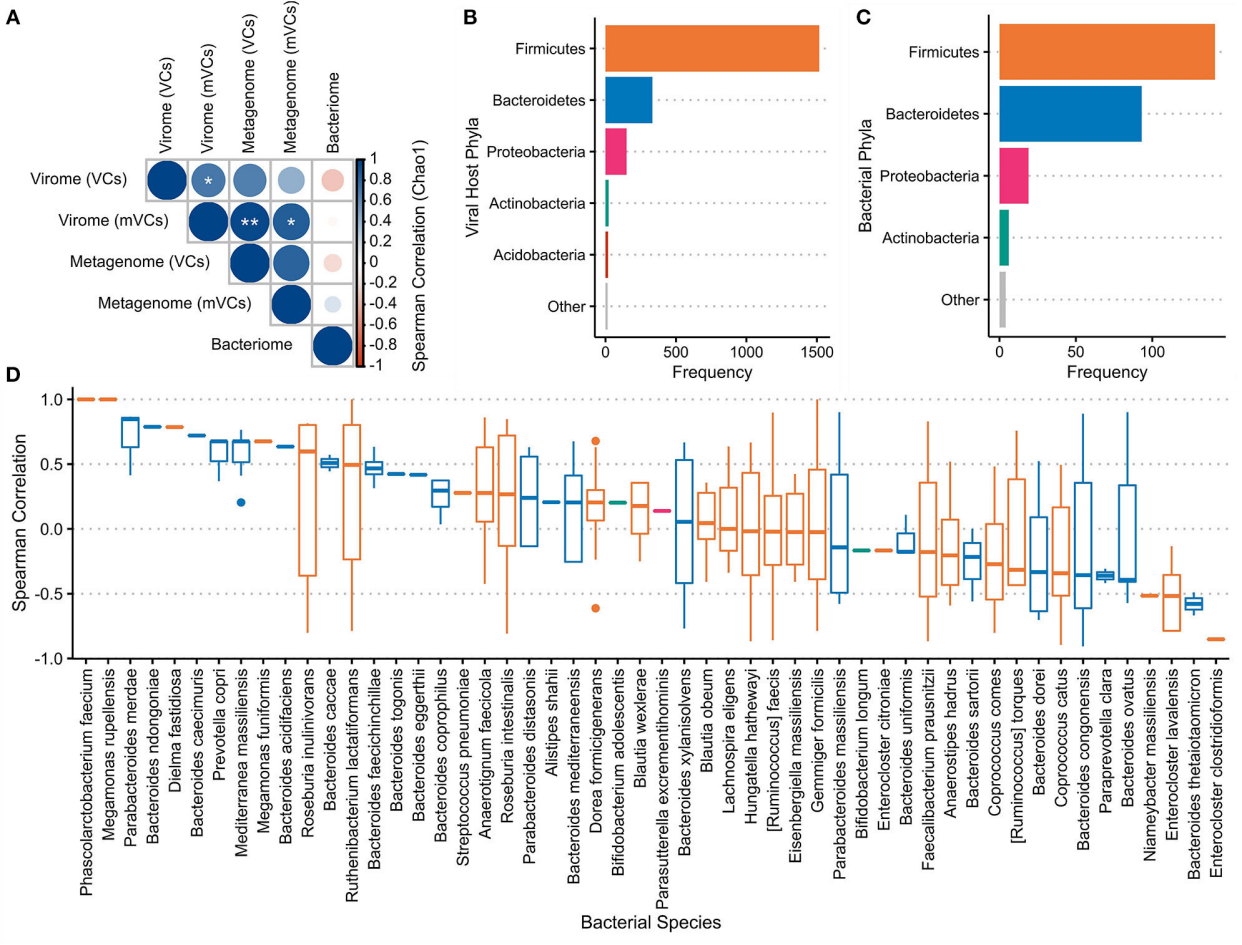
Virome–bacteriome relationships at the mucosal–luminal interface. **(A)** Chao1 indices were used to calculate the alpha-diversity of each sample's bacterial and viral communities, with the latter measured with both virome-derived viral contigs (VC) and metagenome-derived viral contigs (mVC). Spearman correlations between these alpha-diversities were calculated and shown here. *marks a false discovery rate (FDR)-corrected *p*-value of <0.05; ** <0.01. Alpha-diversities for each sample were plotted in Supplementary Figure 7. **(B)** Putative bacterial hosts, mainly representing Firmicutes, Bacteroidetes, and Proteobacteria, were assigned to 2,056 of the 2,511 VCs and plotted in comparison with **(C)** the number of bacterial species in each phylum identified in the bacteriome. **(D)** Fifty-two bacterial species that were present in the metagenome were identified as the most likely host for 411 VCs (excluding Subject C). The Spearman correlation between each VC and associated host species across all samples was plotted. Each boxplot is colored by the bacterial host phyla as presented in panels **(B,C)**.

The Chao1 index was used to measure the alpha-diversity in each sample's virome and bacteriome (Supplementary Figure 7). Spearman correlations were performed between these datasets (Figure 5A). These results show a trend toward positive correlations between the alpha-diversities of all viral communities and inverse correlations between the virome and the bacteriome.

WIsH was used to assign each VC with its most likely host among a reference set of 9,523 bacterial genomes; 2,056 of 2,511 VCs (81.9%) were assigned a putative host. Of these annotated VCs, Firmicutes (73.8%), Bacteroidetes (16.2%), and Proteobacteria (7.25%) were the most predominant bacterial host phyla (Figure 5B), corresponding to the bacterial species observed through whole metagenomic sequencing (Figure 5C). Fifty-two of 593 unique predicted hosts were detected in the annotated bacteriome; these strains were the putative targets of 426 VCs. Spearman correlations between the observed VC and its putative host were calculated across the seven paired samples (411 VCs and 52 hosts) that were subjected to both virome and whole metagenomic sequencing (Figure 5D). Overall, Spearman correlations between VC–host pairs were significantly higher than correlations between VCs and the other 51 non-paired strains (*p* = 2.67e−5).

## Discussion

### The Mucosal–Luminal Interface Enables Site-Specific Study of the Human Gut Virome

Research in the human gut virome is still in its early years, lagging well-behind its bacterial counterpart. Like its predecessor, early virome studies have worked to solve similar challenges, namely, the need for reproducible, standard protocols for both laboratory, and bioinformatic techniques. These developments have led to the necessary exploratory phase, where the virome is being characterized in various states of human health and disease, from infancy to advanced age, and in its relation to diet, drugs, and disease (Garmaeva et al., 2019; Shkoporov and Hill, 2019). Our efforts here expand on these new techniques and aim to extend our knowledge of the “gut” virome beyond fecal samples and into the gastrointestinal tract.

We demonstrate that the MLI is a promising sample type for studying the virome in specific regions accessible to colonoscopy sampling. We have optimized a reproducible virome protocol for this sample type, which removes contaminant host and bacterial nucleic acids and has low background noise (no amplification from a negative control). An exogenous phage added in at known titers could be reliably quantified, while most virome sequencing reads could be aligned to previously sequenced viral populations. The assembly of filtered VCs represented ~94% of all virome sequences in this dataset and could be used for the analysis of viral communities with greater discerning capability than existing databases. Furthermore, this protocol was optimized in subjects with Crohn's disease at various stages in their disease history, demonstrating applicability to clinically relevant conditions.

### Characterizing the Human Colonic Virome

Through our virome sequencing efforts at the MLI, we were able to estimate viral load, quantify virome diversity, and compare viromes between sites and subjects. We estimated total viral loads at the PC of Subjects A and B to be 6.21 ± 0.13 × 10^8^/ml and 1.80 ± 0.31 × 10^8^/ml, respectively. This represents an order of magnitude fewer viral particles than in stool using similar purification, MDA, and sequencing techniques (Shkoporov et al., 2018). The decreased viral load could reflect the sampling methodology and/or the decreased microbial load in the PC compared with stool (Sender et al., 2016). Further characterization of the viral load across the gastrointestinal tract in healthy and disease states using quantitative virome sequencing approaches would provide important context for future virome and metagenome studies.

We identified 2,511 VCs across nine virome samples. Over 60% of VCs could be annotated by Demovir, with *Caudovirales* representing nearly all classified VCs. Thirty-eight VCs were classified as *Anelloviridae* and *Microviridae*, which represent families of ssDNA viruses that are preferentially amplified by MDA (Kim and Bae, 2011). These were enriched compared with two *Microviridae* identified among mVCs.

We observed an average of 648 VCs per sample that were present at ≥75% horizontal coverage; most of these were subject-specific, while 435 VCs across four subjects were also site-specific. Beta-diversity analysis showed that intrasubject viromes were similar but not identical, demonstrating a higher Bray–Curtis distance than same-sample replicates and highlighting virome diversity across the gastrointestinal tract. Intersubject viral diversity was also higher than bacterial diversity. We did not identify a core virome, which has been previously reported in healthy adult cohorts (Manrique et al., 2016; Shkoporov et al., 2019); this result could reflect higher viral diversity in inflammatory bowel disease and/or a pediatric population. However, the specific impact of Crohn's disease and other clinical metadata on VC diversity or composition were not examined due to the low number of subjects and could be the focus of future studies.

### Interpreting Viral Sequences in Whole Metagenome Data

The MLI samples yield sufficient microbial content for whole metagenomic sequencing, which provides additional context for virome studies that is missed when only using 16S rRNA gene sequencing. While host sequencing proportions averaged 40% per sample and was as high as 90% in one sample, the MLI still offers a significant improvement from intestinal biopsies, which yields >95% host DNA (Zhang et al., 2015). Our data suggest that this variation could be due to disease activity: host DNA was higher in treatment-naïve patients at diagnosis (Subjects D and E: 25.2–90.0%) than during remission (A and B: 0.362–2.63%), in line with what other studies have reported in stool (Lewis et al., 2015).

We were able to use the shotgun metagenomic sequencing reads to perform VC assembly from the whole metagenome, compare viral populations, and perform virome–bacteriome analysis at a species level. We assembled 3,122 mVCs, though only 28% of contigs could be annotated. The reduced classification compared with the virome could be due to the shorter contig length or potential misassembles including bacterial reads that were not filtered out, suggesting the need for further decontamination tools. Like the VCs, most mVCs were *Caudovirales*, though small numbers of *Phycodnaviridae, Mimiviridae, Iridoviridae*, and *Herpesviridae* were enriched compared with the VCs. These eukaryotic viruses could represent viruses that are selected against in the VLP extraction protocol (such as filters that exclude larger *Megavirales*) or possible false annotations with Demovir (Sutton et al., 2020).

An average of 6.24% and a high of 10.9% of host-removed reads in each metagenome sample could align to the assembled mVCs, decreasing to a mean of 4.35% and maximum of 9.94% of reads aligning to VCs. The significance of VC alignments in whole metagenome data is not well-understood, limited by the lack of viral databases and the predominant use of 16S rRNA gene sequencing to characterize the bacteriome, even in virome–metagenome studies. Several bioinformatic approaches have been developed to mine existing whole metagenome data for viruses, including VirSorter (Roux et al., 2015), VirFinder (Ren et al., 2017), virMine (Garretto et al., 2019), PhagePhisher (Hatzopoulos et al., 2016), and others (Paez-Espino et al., 2017). A reanalysis of a human microbiome gene catalog that originally attributed 0.1% of its contents to both eukaryotes and viruses had 1.31–38.43% of contigs predicted as viral; the authors attributed this discrepancy to prophages (Garretto et al., 2019). Efforts to infer phage attributes include alignments to well-characterized phages and prophages, searches for integrases and transposes, and tests for circularity; yet it remains difficult to interpret the presence of phage assemblies and viral alignments from short-read metagenomic studies. Given the dynamic potential for bacteriophages to be present as integrated prophages, extrachromosomal elements, intracellular phages, extracellular free or membrane-bound phages, and other forms (Hobbs and Abedon, 2016), sequencing approaches will need to be paired with other fields of study to contextualize these results.

We were unable to meaningfully detect our spike-in phage through whole metagenomic sequencing. This result can be attributed to the fact that our microbiome extraction methods do not aim to retain or lyse viral particles. Alternatively, or in concert with kit limitations, the quantity of free phage DNA at physiological concentrations may be outcompeted by microbial DNA to a degree that renders deep shotgun sequencing ineffective for detection. These findings should be further tested with a diverse range of phages and additional samples with various DNA extraction methods; regardless, VC alignments in our whole metagenomic sequences are unlikely to represent free phages. These reads are thus more likely to represent prophages or other forms of intracellular phages, while the virome sequencing reads would exclude prophages and could explain the alignment gaps between VCs and mVCs. Both our clustering efforts and beta-diversity analysis demonstrate that the VCs and mVCs represent distinct viral communities with some overlap represented by 251 clusters containing 837 contigs. Bray–Curtis distances suggest that the viral portion of the whole metagenome may be more conserved than the VLP-enriched virome, which could include a community of prophages that are only induced under specific conditions.

Importantly, these results indicate that virome studies that employ metagenome-mining techniques should be interpreted differently than VLP-enriched viromes, echoed by recent analysis by Gregory et al. (2020). We also similarly report an increased number of VCs identified in the whole metagenome with a decreased average contig length when comparing paired samples. Differences between these two datasets can only be investigated when both virome-focused and whole metagenomic sequencing are performed in parallel, an analysis that has been rarely performed in virome studies (Gregory et al., 2020). These comparisons are made easier by MLI sampling, though these analyses will need to consider biases in each methodology, including the choice of extraction kit and use of amplification techniques (e.g., MDA).

### Virome–Bacteriome Interactions

We observed inverse alpha-diversities between viral and bacterial communities, which was previously observed in the infant virome (Lim et al., 2015). Lim et al. attributed this relationship to dynamic changes in the developing infant gut microbiome; whether this effect is also demonstrated in these patients will require a control cohort of subjects without Crohn's disease.

We also identified specific VC–host species pairs based on WIsH predictions against a large set of reference bacterial genomes. We focused on VC–host pairs involving bacterial strains also identified in our bacteriome. For 411 VC–host pairs involving 52 unique bacterial species, Spearman correlations across seven samples were calculated. Positive correlations reflect stable host–phage interactions (Reyes et al., 2010; Lim et al., 2015), suggestive of “piggyback-the-winner” relationships that have been proposed for the mucosa-associated virome (Silveira and Rohwer, 2016); negative correlations suggest predatory–prey relationships. We were able to visualize these potential interactions with species-level resolution. Compared with unpaired VC–host interactions (i.e., a null comparison), we observed significantly higher Spearman correlations in our VC–host pairs (*p* = 4.78e−6). This result suggests that VC–host pairs in the MLI microbiome tend to be positively correlated. Interpretation of these data is limited by sample size and complicated by host inflammation; further studies are required to explore these interactions and investigate how virome–bacteriome dynamics are affected by additional stresses like host disease. Additionally, incorporation of CRISPR spacers and other annotation tools could strengthen these analyses.

### Protocol Limitations

While we were able to characterize the colonic MLI virome, we were unable to recover viruses from the TI. This limitation may be due to a lower viral load, though a DC sample also lacked sufficient DNA; further optimization of techniques or a higher biomass may be required.

Our protocol employed MDA to increase DNA input for virome sequencing. While frequently used, MDA approaches can skew the observed viral community, selecting for small, circular ssDNA viruses (Kim and Bae, 2011). Newer approaches including alternative linker amplification or tagmentation may be implemented within our protocol to reduce this bias (Roux et al., 2016). Like other virome sequencing efforts, we also did not attempt to characterize enveloped or RNA viruses. RNA viruses tend to represent transient, plant pathogens that are less likely to be involved in human health (Zhang et al., 2005). Additionally, enveloped RNA viruses encompass many human viruses including severe acute respiratory syndrome coronavirus 2 (SARS-CoV-2); these human pathogens tend to be comparatively well-characterized and/or have existing tools to enable their direct study. Moreover, outside of viral infections, eukaryotic viruses are typically found in low abundance in the human gut (Garmaeva et al., 2019); their role in microbiome-implicated human diseases like inflammatory bowel disease has also been suggested to be limited (Tokarz et al., 2019), though potential eukaryotic virome signatures have been reported (Ungaro et al., 2018).

### Future Directions

We demonstrate that our protocol enables the site-specific study of the human gut virome in the context of the whole metagenome at the MLI. Future research can apply these techniques to further investigate many of the hypotheses discussed here, while also examining the virome in site-specific pathologies like Crohn's disease.

Many key questions about the human gut virome remain, including whether a core healthy human virome exists and whether phage treatments could be a viable approach in microbiome modulation therapies. These questions cannot be fully answered with stool alone. A focused effort to characterize the virome along the length and cross section of the gastrointestinal tract is required to provide a higher-resolution understanding of virome–bacteriome–host interactions. Studying the MLI virome is one step forward in these efforts to develop a more comprehensive model of the human microbiome.

## Data Availability Statement

The datasets presented in this study are available online from NCBI databases under BioProject: PRJNA645218 (https://www.ncbi.nlm.nih.gov/bioproject/645218).

## Ethics Statement

The studies involving human participants were reviewed and approved by the Research Ethics Board of the CHEO Research Institute. Written informed consent to participate in this study was provided by the participants' legal guardian/next of kin.

## Author Contributions

AY performed all experiments, sequencing, and data analysis and wrote the initial manuscript. DM recruited patients, performed endoscopy on patients, and provided access to relevant clinical metadata. AY and AS designed the experiments. JB provided input on the bioinformatic analysis and manuscript. All authors reviewed and provided comments on the final manuscript.

## Conflict of Interest

AS and DM are co-founders of MedBiome, a clinical microbiomics company. The remaining authors declare that the research was conducted in the absence of any commercial or financial relationships that could be construed as a potential conflict of interest.

## Supplementary Material

Supplementary Figure 1 Summary of virome and whole metagenome DNA extraction and sequencing protocols. Viromes and whole metagenomes were extracted from mucosal-luminal interface samples. Virus-like particle purification and the removal of remaining bacterial cells were required for efficient virome sequencing. The full protocol is described in Materials and Methods. VLPs: virus-like particles; PEG: polyethylene glycol; CTAB: cetyltrimethylammonium bromide.

Supplementary Figure 2 Summary of bioinformatic pipeline and subsequent analysis. Sequencing reads were first quality filtered and subjected to host-read removal. Both virome and metagenome sequencing reads were then assembled into contigs which were subjected to a viral contig identification pipeline. Sequencing reads could then be mapped to these contigs for further analysis. Bacteriome annotation of the whole metagenome sequencing reads was also performed. Full details and programs are described in Materials and Methods. VC, virome-derived viral contigs; mVC, metagenome-derived viral contigs; B, bacteriome; ORFs, open reading frames; COG, Clusters of Orthologous Groups of proteins.

Supplementary Figure 3 Virome sequencing reads matching exogenous phage are linearly correlated with spike-in phage titers. An exogenous phage, NCTC 12673, was added to mucosal-luminal interface aspirates from the proximal colon (PC) of subjects A and B at concentrations of 10^5^, 10^6^, and 10^7^ pfu ml^−1^. Virome sequencing reads were mapped to the phage genome. The proportion of reads aligning to NCTC 12673 were plotted against the phage titers, showing a linear relationship (*R*^2^ > 0.99).

Supplementary Figure 4 Viral contigs at the colonic mucosal-luminal interface (subsetted dataset). Prior to viral contig mapping, virome sequencing reads for each sample were randomly subsetted to two million reads to represent an even sequencing depth. **(A)** All viral contigs (VC) were plotted by their maximum observed abundance (RPKM-adjusted) vs. length. VCs were colored by the number of subjects where the contig was observed at ≥75% horizontal contig coverage. **(B)** The number of contigs present at ≥75% horizontal coverage was plotted for each sample, shaded by whether the contig was only observed in that sample, that subject, or in two or more subjects (“shared”). PC, proximal colon; DC, distal colon.

Supplementary Figure 5 Clustering of virome-derived and metagenome-derived viral contigs. Viral contigs from virome (VC) and metagenome (mVC) datasets were pooled and clustered using vConTACT2. Contig networks were plotted using Cytoscape 3.7.2 using its default Perfuse Force Directed Layout using vConTACT2-derived edge weights. Outliers, singletons, and doubletons were excluded from the network visualizations. Contigs are colored by viral annotations from Demovir for each network, while the combined network is also shown colored by VCs and mVCs in the right panel.

Supplementary Figure 6 Beta-diversity of the mucosal-luminal interface virome and bacteriome. Bray-Curtis distances between samples were calculated using the assembled viral contigs (VC) in the virome, metagenome-derived viral contigs (mVC) in the whole metagenome, and bacterial taxa (B) in the whole metagenome. **(A)** Principal coordinate analyses were plotted for each dataset. **(B)** Bray-Curtis distances were compared between subjects and within subjects for each dataset. ^*^indicates an FDR-corrected *p*-value of < 0.05 against every other subgroup; + indicates significance against all other comparisons except *a*; *a* indicates significance against all other inter-subject comparisons except +; *b* indicates significance against inter-subject comparisons only.

Supplementary Figure 7 Alpha-diversity of the mucosal-luminal interface virome and bacteriome. Using the Chao1 index, alpha diversity was measured using the relative abundance of viral contigs (VCs) and metagenome-derived viral contigs (mVCs) in the virome, VCs, and mVCs in the whole metagenome, and bacterial taxa in the metagenome (B). For each dataset, read counts were first subsetted to the sample with the lowest number of mapped reads (Virome / VC: 1,877,966; Virome mVC: 528,240; metagenome / VC: 5,358; metagenome / mVC: 5,867; bacteriome: 38,711).
