# Supplementary figures and images for: Virome Sequencing of the Human Intestinal Mucosal–Luminal Interface
Source: Front Cell Infect Microbiol. 2020 Oct 22;10:582187. doi: 10.3389/fcimb.2020.582187 (PMC7642909; doi:10.3389/fcimb.2020.582187)

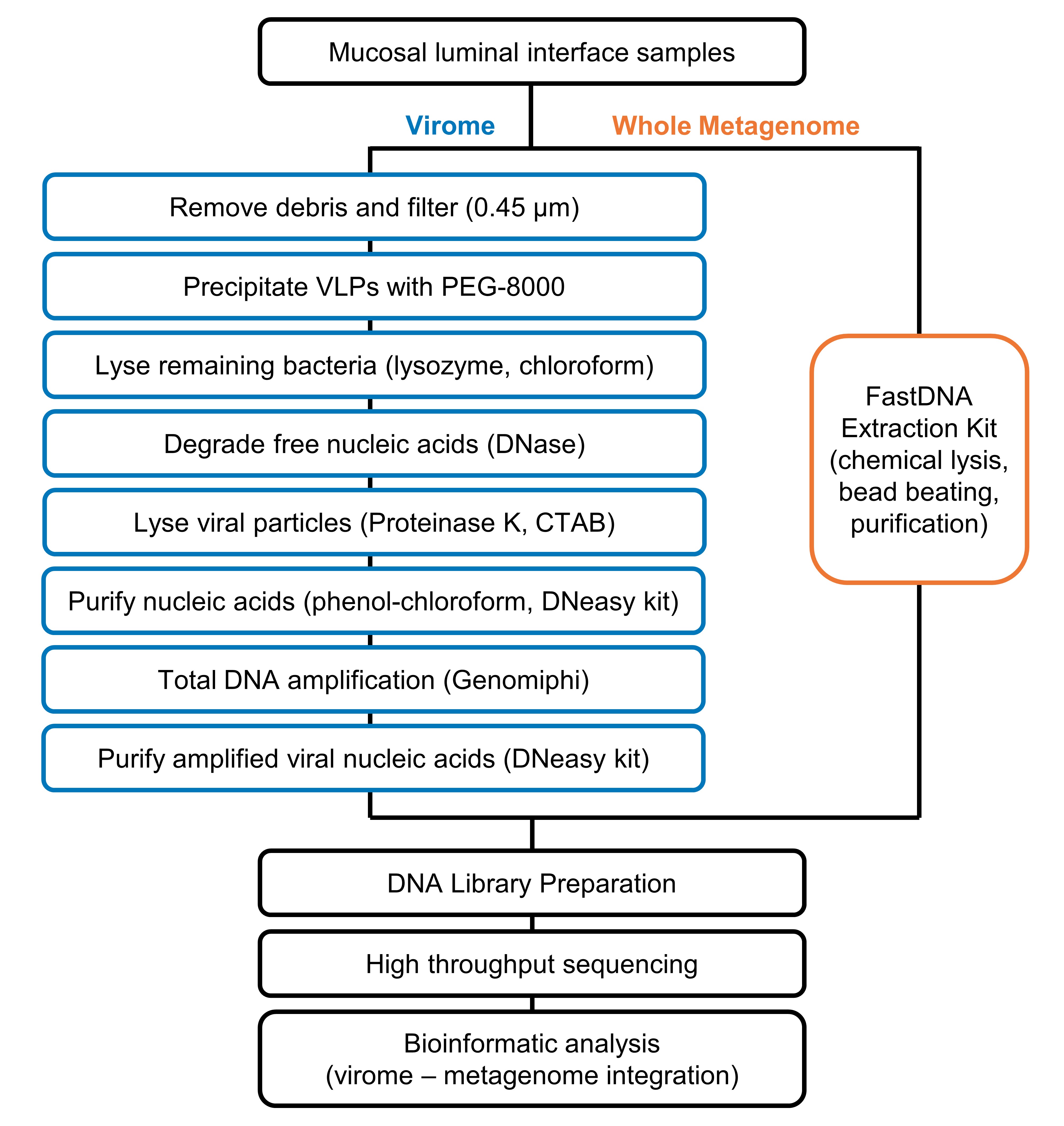

Supplement: Supplementary Figure 1 — Summary of virome and whole metagenome DNA extraction and sequencing protocols. Viromes and whole metagenomes were extracted from mucosal-luminal interface samples. Virus-like particle purification and the removal of remaining bacterial cells were required for efficient virome sequencing. The full protocol is described in Materials and Methods. VLPs: virus-like particles; PEG: polyethylene glycol; CTAB: cetyltrimethylammonium bromide. [file Image_1.JPEG]

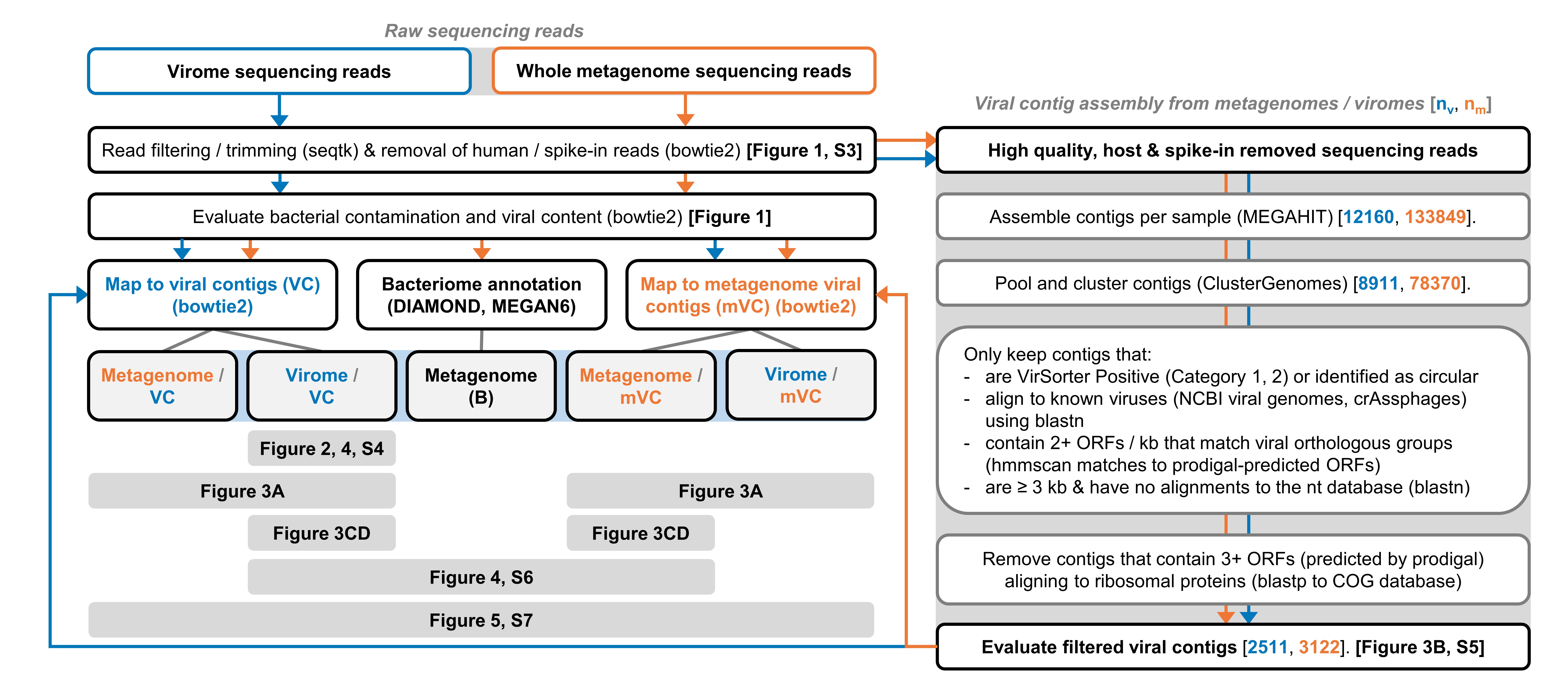

Supplement: Supplementary Figure 2 — Summary of bioinformatic pipeline and subsequent analysis. Sequencing reads were first quality filtered and subjected to host-read removal. Both virome and metagenome sequencing reads were then assembled into contigs which were subjected to a viral contig identification pipeline. Sequencing reads could then be mapped to these contigs for further analysis. Bacteriome annotation of the whole metagenome sequencing reads was also performed. Full details and programs are described in Materials and Methods. VC, virome-derived viral contigs; mVC, metagenome-derived viral contigs; B, bacteriome; ORFs, open reading frames; COG, Clusters of Orthologous Groups of proteins. [file Image_2.JPEG]

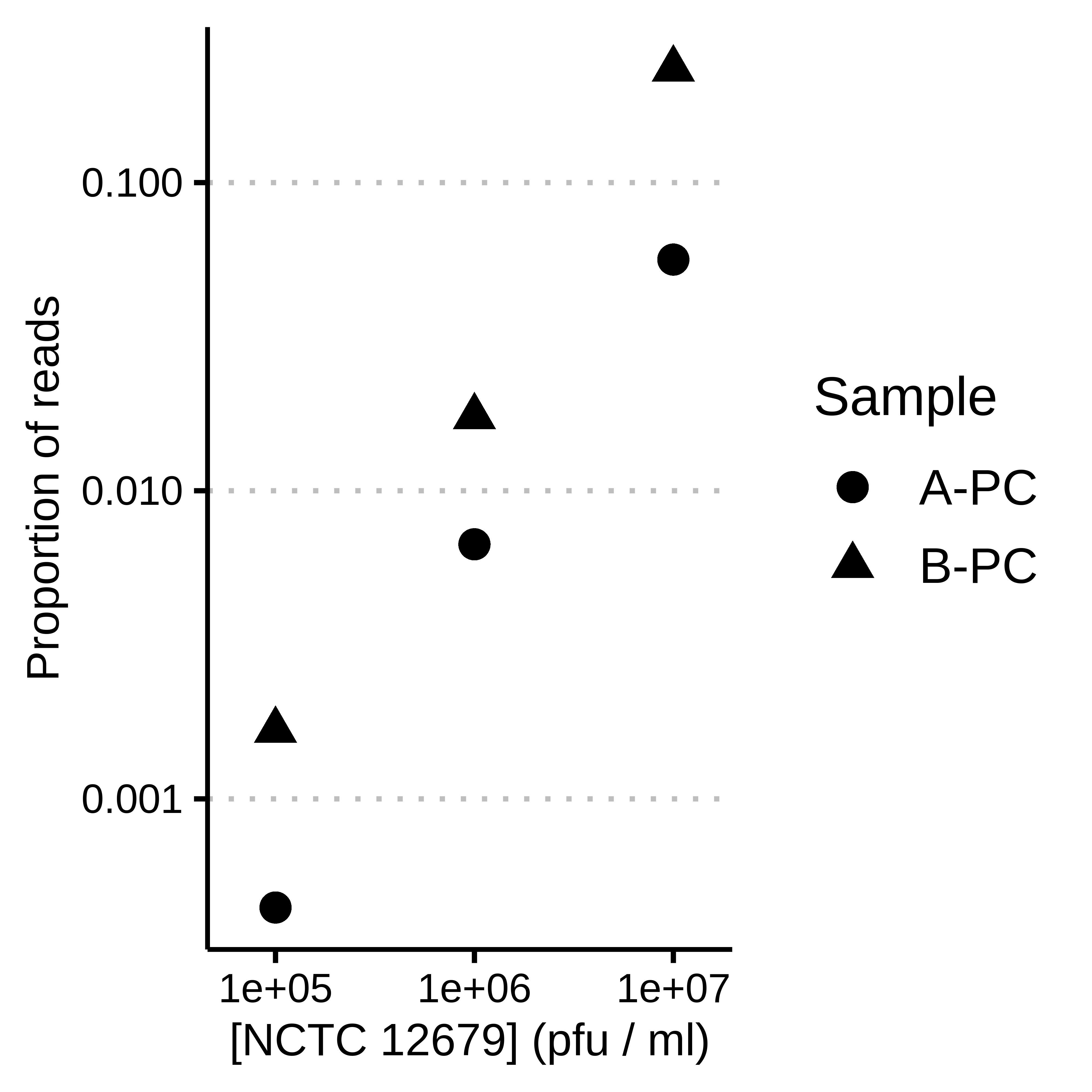

Supplement: Supplementary Figure 3 — Virome sequencing reads matching exogenous phage are linearly correlated with spike-in phage titers. An exogenous phage, NCTC 12673, was added to mucosal-luminal interface aspirates from the proximal colon (PC) of subjects A and B at concentrations of 105, 106, and 107 pfu ml−1. Virome sequencing reads were mapped to the phage genome. The proportion of reads aligning to NCTC 12673 were plotted against the phage titers, showing a linear relationship (R2 > 0.99). [file Image_3.JPEG]

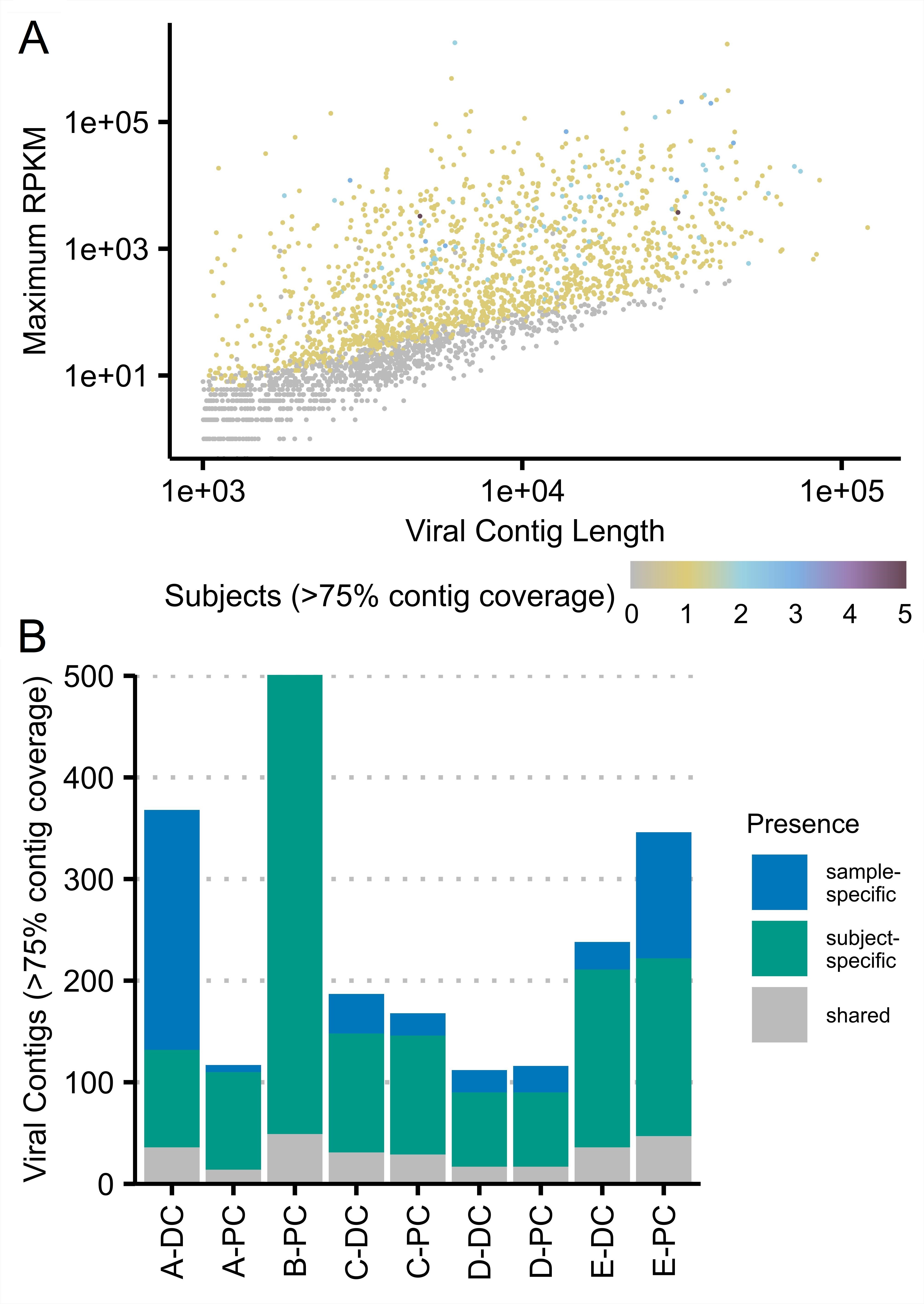

Supplement: Supplementary Figure 4 — Viral contigs at the colonic mucosal-luminal interface (subsetted dataset). Prior to viral contig mapping, virome sequencing reads for each sample were randomly subsetted to two million reads to represent an even sequencing depth. (A) All viral contigs (VC) were plotted by their maximum observed abundance (RPKM-adjusted) vs. length. VCs were colored by the number of subjects where the contig was observed at ≥75% horizontal contig coverage. (B) The number of contigs present at ≥75% horizontal coverage was plotted for each sample, shaded by whether the contig was only observed in that sample, that subject, or in two or more subjects (“shared”). PC, proximal colon; DC, distal colon. [file Image_4.jpg]

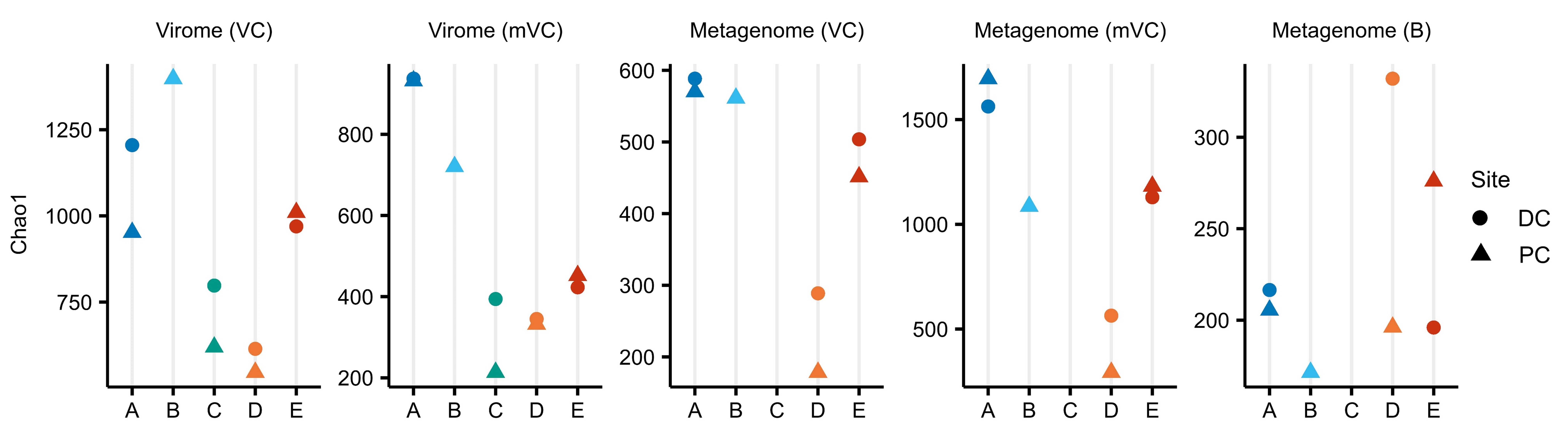

Supplement: Supplementary Figure 7 — Alpha-diversity of the mucosal-luminal interface virome and bacteriome. Using the Chao1 index, alpha diversity was measured using the relative abundance of viral contigs (VCs) and metagenome-derived viral contigs (mVCs) in the virome, VCs, and mVCs in the whole metagenome, and bacterial taxa in the metagenome (B). For each dataset, read counts were first subsetted to the sample with the lowest number of mapped reads (Virome / VC: 1,877,966; Virome mVC: 528,240; metagenome / VC: 5,358; metagenome / mVC: 5,867; bacteriome: 38,711). [file Image_7.JPEG]
